# Urban Seismic Network Based on MEMS Sensors: The Experience of the Seismic Observatory in Camerino (Marche, Italy)

**DOI:** 10.3390/s22124335

**Published:** 2022-06-08

**Authors:** Giovanni Vitale, Antonino D’Alessandro, Andrea Di Benedetto, Anna Figlioli, Antonio Costanzo, Stefano Speciale, Quintilio Piattoni, Leonardo Cipriani

**Affiliations:** 1 National Earthquake Observatory, Istituto Nazionale di Geofisica e Vulcanologia, 00143 Rome, Italy; antonino.dalessandro@ingv.it (A.D.); antonio.costanzo@ingv.it (A.C.); stefano.speciale@ingv.it (S.S.); 2 Department of Matematics and Computer Scienze, University of Palermo, 90123 Palermo, Italy; andrea.dibenedetto@unipa.it; 3 Department of Earth and Sea Sciences, University of Palermo, 90123 Palermo, Italy; anna.figlioli@unipa.it; 4 Office of Public Works, Maintenance, Environment and Seismic Reconstruction, Municipality of Camerino, 62032 Camerino, Italy; quintilio.piattoni@comune.camerino.mc.it; 5 School of Architecture and Design, University of Camerino, 63100 Ascoli Piceno, Italy; leonardo.cipriani@unicam.it

**Keywords:** low-cost accelerometer, earthquake monitoring system, early warning system, shake map, observatory sensors, micro-electro-mechanical system (MEMS)

## Abstract

Urban seismic networks are considered very useful tools for the management of seismic emergencies. In this work, a study of the first urban seismic network in central Italy is presented. The urban seismic network, built using MEMS sensors, was implemented in the urban district of Camerino, one of the cities in central Italy with the greatest seismic vulnerability. The technological choices adopted in developing this system as well as the implemented algorithms are shown in the context of their application to the first seismic event recorded by this innovative monitoring infrastructure. This monitoring network is innovative because it implements a distributed computing and statistical earthquake detection algorithm. As such, it is not based on the traces received by the stations from the central server; rather, each station carries out the necessary checks on the signal in real time, sending brief reports to the server in case of anomalies. This approach attempts to shorten the time between event detection and alert, effectively removing the dead times in the systems currently used in the Italian national network. The only limit for an instant alarm is the latency in the tcp/ip packages used to send the short reports to the server. The presented work shows the infrastructure created; however, there is not enough data to draw conclusions on this new early warning approach in the field, as it is currently in the data collection phase.

## 1. Introduction

When a strong earthquake occurs, the loss of human life depends primarily on the intensity of the shaking, the vulnerability of the buildings, and the effectiveness of the rescue operations in the immediate post-quake period. The only variable on which it is possible to operate in a short time with limited expenses is the effectiveness of relief operations in the immediate post-earthquake. A local emergency management centre can, through timely and targeted actions in the immediate post-earthquake period, reduce the operations time by means of an Urban Seismic Network (USN). A USN is a highly dense grid of accelerometric stations installed within an urban centre [1,2]. Local emergency management personnel can use information provided by the USN, such as immediate alert and post-earthquake information summarized in maps of ground motion parameters, to decide on action priorities in order to minimize the loss of human life by optimally managing the available resources and increasing the safety of operations [3]. In recent years, many urban seismic observatories have been created around the world: [4,5,6,7,8,9,10,11,12]. This was possible thanks to important developments in the field of MEMS (Micro-electro-mechanical systems) technology, especially MEMS accelerometers. MEMS accelerometers are electromechanical devices able to measure static or dynamic accelerations, and are currently widely used in strong motion seismology [13,14,15]. In recent years, seismic activity in the Central Apennines has increased considerably, and with it the interest of the scientific community. The Italian nation has not experienced a very large earthquake for more than thirty-five years; the last, in Irpinia, measured a 6.9 Mw. In 2016, a seismic sequence occurred in the Marche region which caused more than 300 deaths and razed many small towns, including Camerino, which was 30 km away from the seismogenic source. This was not the only event; in 2009, there was another seismic sequence that affected the area around the city of Aquila, with a magnitude of 6.3 Mw. The central Apennines are characterized by north-east-verging thrust-propagation folds involving Mesozoic–Tertiary sedimentary successions [16], characterized predominately by an extensional dynamic of spreading above the Calabrian Arc [17]. In fact, due to the major events that occurred during the 2016 seismic sequence, all of these returned focal mechanisms of normal activity. Among the various faults present in the area under study, the one that characterized the seismic events from 2016 onwards was the fault passing through Monte Vettore (System M.Bove–M.Vettore).

**Figure 1:**
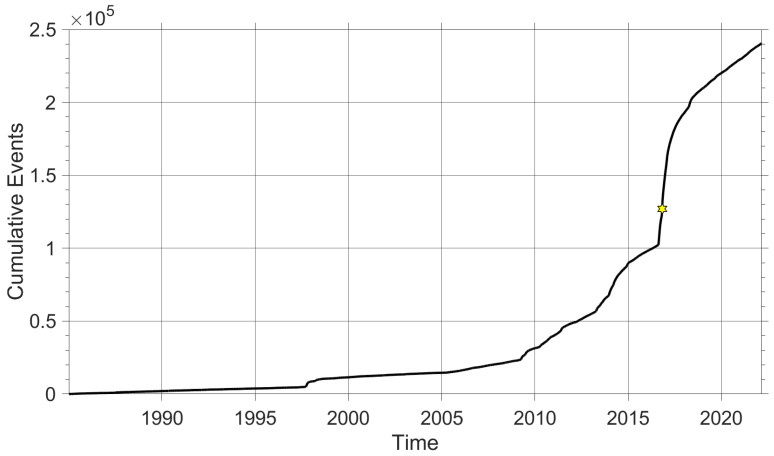
Representation of cumulative curve of events, with a time range from 1985 to 2022. The star represents the Norcia M6.5 earthquake, which was the strongest event of the 2016/2017 central Italy seismic sequence.

**Figure 2:**
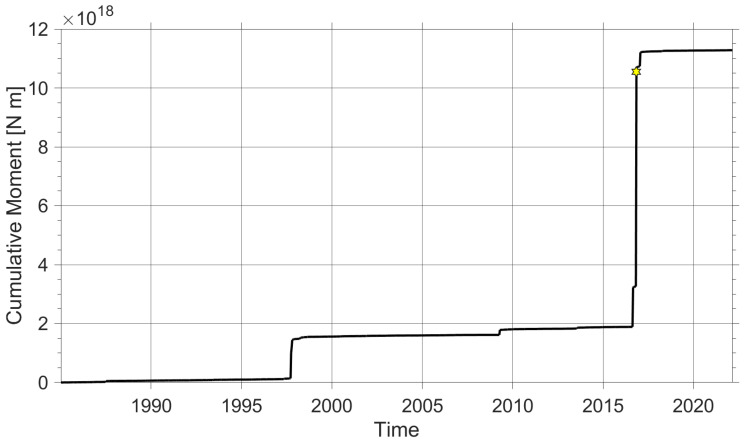
Representation of the cumulative moment curve in the time range from 1985 to 2022. The star represents the Norcia M6.5 earthquake, which was the strongest event of the 2016/2017 central Italy seismic sequence.

To understand the historical moments of seismic crisis in the Central Apennines, two graphs are plotted, show in Figure 1 and Figure 2. More specifically, Figure 1 shows the cumulative curve of events dating back to the beginning of the recording of the first seismic events with the permanent stations of the INGV (Istituto Nazionale di Geofisica e Vulcanologia) up to the present, that is, 28 February 2022. The graphic shows a significant increase in events, increasing almost exponential. Figure 2 represents the cumulative moment, present as two significant step that characterize a seismic sequence. The dataset used to plot the seismic activity of the area under study was downloaded from the official website (http://terremoti.ingv.it/, accessed on 20 April 2022) of the INGV [18]. In this work, we want to introduce the first Urban Seismic Network developed in central Italy, in particular in Camerino, a town in the ARCH project [19]. First, the principles of urban seimsic monitoring network are explained, including topology and the seismic stations technology (Section 2 and Section 5). Following (Section 4), the algorithms implemented on board and on the server are explained. Section 3 deals with the validation of the performance of low-cost accelerometers by means of a professional calibration system. This is an important aspect when considering the use of non-professional sensors. In [20], the authors studied the main sources of error of a low-cost three-axis MEMS accelerometer using the Power Spectral Density (PSD) and Allan Variance (AV) techniques. In Section 6, the Camerino Network is described in detail. Finally, in Section 7, an example of an earthquake recorded by the Camerino urban seismic network is shown and conclusions are drawn.

## 2. MEMS Stations

The software system of ARES station is composing of data acquisition using mSEED format (this is standardized protocol for the exchange of earthquake data used by seismologists worldwide [21]) and data transmission with Ring-Server. The other element is optional; an NTP (Network Time Protocol) server increases time precision using GPS (Global Position System) or internet information, and slarchive generates a local storage of data (for details, please refer to [20]).

Data transmission makes it possible to use LTE devices. The LTEs chosen for the project are the same that have been used in and extensively tested for Italian national seismic network stations. It is recommended to use an LTE device with two slots for SIM chips in order to ensure network redundancy, as well as two Ethernet ports to permit future expansion of the network with other devices. These must have a robust protection system, with various passwords and an IP filter built in. The possibility of protecting the devices with an IP filter allows for excellent IT security, as only devices with set IPs can interact with the system. Through these apparatuses, is possible to achieve near real-time data acquisition, management, and maintenance of the network and equipment. The SIM cards used for data transmission were M2M cards, and the NAT service was active. The accelerometer integrated in the MEMS model 1043_0 (https://www.phidgets.com/?&prodid=31, accessed on 20 April 2022) can operate in two modes: a high sensitivity mide (“Precision”) for accelerations in the range ±2 g, and a “Backup” mode for higher accelerations. Because the Backup mode is activated only for accelerations that are hard to imagine due to an earthquake, in the following description of the specifications provided in the Table 1 above reference is only made to those relating to the Precision mode. Table 1 shows the specifications of the triaxial accelerometer in Precision mode according to the data sheet provided by Phidgets.

In particular, the signal output is in a 16-bit format with a maximum sampling frequency of 1000 Hz. This sensor is suitable for possible use in the field of applied seismology (for details please refer to [20]). The elements that compose the seismic station can be replaced with components or devices that perform the same functions while having different technical characteristics and better performance. This represents a strong point of the monitoring system, as there is the possibility of adapting the seismic station to different needs and installation conditions.

## 3. Sensor Validation

### 3.1. Calibration System

**Figure 3:**
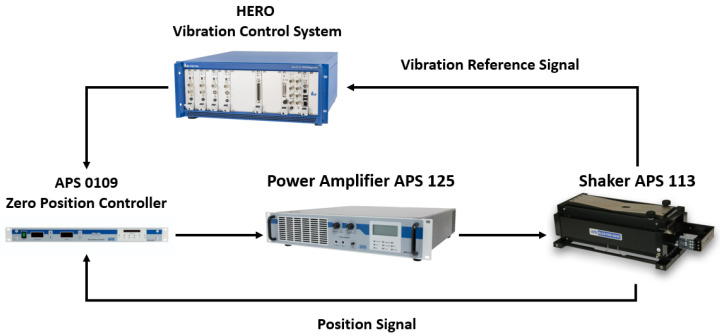
Block diagram of calibration system.

The APS 113-AB ELECTRO-SEIS (APS Dynamics, Inc. of SPEKTRA GmbH, Dresden, Germany) is an electrodynamic actuator, and is used to generate force on a system for calibration and evaluation of accelerometers and other motion transducers. In particular, it is employed for low frequency excitation of such devices. Drive power is obtained from an APS 125 low frequency power amplifier. The maximum overhung load that can be used is 1.5 kg concentrated at the mounting point. Zero position control is possible with APS 0109, as it automatically controls the position of a vibration exciter irrespective of its load. The harmonic content of the output is very small, as heavy negative feedback is used. The instrument can tolerate temperature and supply line variations while maintaining excellent stability and integrated automatic load compensation. In Figure 3, it is represented by a Block Diagram showing the Calibration System.

### 3.2. Test Protocol

**Figure 4:**
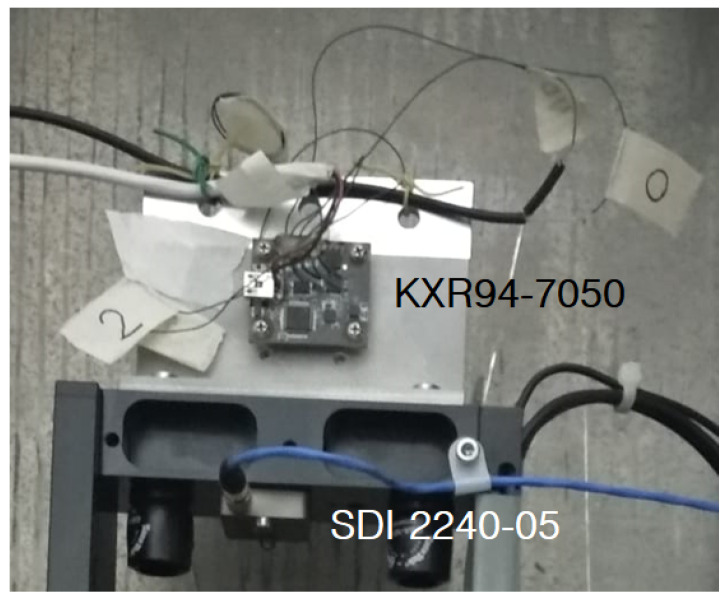
The figure shows the tightening of two sensors in detail; SDI 2240-05 is a sensor (a single-axis capacitive MEMS with high performance) in the calibration system, while KXR94-7050 is the test sensor (a three-axis MEMS with good capacity and performance for seismic engineering engineered and marketed by the Canadian company Phidget under the name 1043_0.

The Phidgets 1043_0 accelerometer used here was engineered with a large number of APIs and libraries to directly manage the data flow. As it does not have an analog output, it was necessary to carry out a circuit analysis in order to understand whether it was possible to obtain the power points and the analog outputs to be supplied to the calibration system. As the component used by Phidgets, the KXR947050 accelerometer, has analogue output conditions on the board, it was possible to obtain the outputs of the three axes and the temperature in analog (Figure 4).

The sensor under testing was fixed with a steel “L” bracket suitably made to mount on the calibration system bracket. The fixing of the sensor under testing on the bracket was made with spacers and bolts supplied by the manufacturer. As an integral part of the calibration apparatus, SDI 2240-05 was fixed by screws in the head of the carriage in the same block in which the test sensor bracket was fixed (Figure 4).

**Figure 5:**
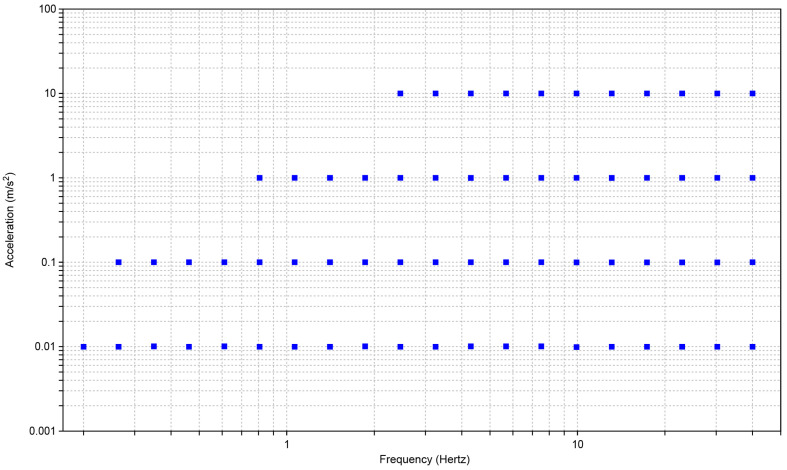
Parameters imposed for setting sinusoidal movements during measuring protocol. The blue squares represent the accelerations with respect to the frequency for each shaking test.

The evaluation of the sensor was carried out by imposing a sinusoidal movement on the system; below, Figure 5 shows values set for the tests.

**Figure 6:**
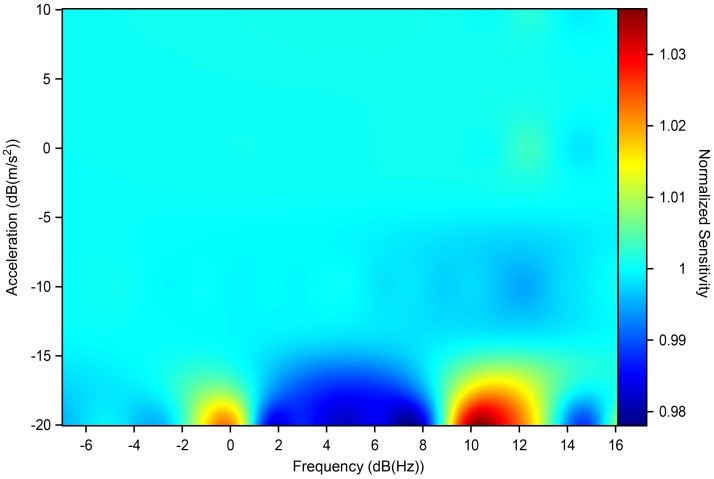
Variation in sensitivity as interpolation of the results obtained by shaking tests.

**Figure 7:**
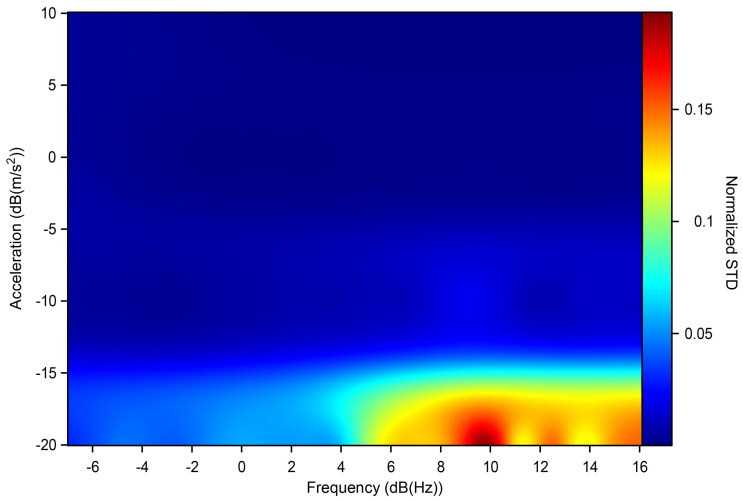
STD of the measurements as interpolation of the values obtained by shaking tests.

The testing was performed in the following way: the system was started with a check test assuming a reference frequency of 8 Hz and a peak acceleration of 1.4142 m/s^2^ for ten cycles. If there were no errors in the test phase, after 30 s the system began to execute the values in Figure 5 for ten consecutive cycles. Between one value and another, the system stopped for 15 s before imposing the new desired values. At the end of the session, the data collected were viewed in a report and exported in .txt format; the report included a graph of the module and the phase of the distortion between the two instruments. In order to perform a statistical analysis of the data obtained, the experiment was carried out five times per axis; below, we show the points obtained and their means, Figure 6 and Figure 7.

The results shown in the figure below are an example of the ten sensors, tested axis by axis (three axes per sensor). It can be seen that the differences between the axes of the same sensor or of other sensors under testing do not show significant variations, which means that the commercial series of sensors chosen has excellent behaviour in the range of interest analysed here. From Figure 6, it can be seen that for very low acceleration values it is very likely that the detected acceleration can be estimated by about ±2%; Figure 7 shows the STD of the measurement sessions performed, indicating that the results obtained are robust and have good repeatability.

## 4. Innovative Distributed Computing for Statistical Earthquake Detection Algorithm

For the purposes of analysis, seismic events are classified into three categories: local, regional, and teleseismic events. This classification allows for recognition of a seismic event in relation to its distance from the seismic station. For every class, there is a pass band frequency interval that identifies the category. For local events, which are rich in high frequencies, the pass band frequency range is 4–40 Hz. In regional events, high frequencies are attenuated, and the pass band frequency is 0.4–4 Hz. Finally, in teleseismic class events, rich in low frequencies, the pass band frequency is 0.1–0.4 Hz. Filtering is performed digitally in real time with a 1 kHz refresh rate. In order to build the structure of the filters, we began with the mathematical model of a simple analogue band pass filter, on which the bilinear transform was then applied to switch from continuous to discrete time. The formula used for the calculation of the filtered waveform is as follows:

(1) $$Y_{\left(k\right)}=K_{1}Y_{\left(k-2\right)}+K_{2}Y_{\left(K-1\right)}+K_{3}\left(U_{\left(k\right)}-U_{\left(k-1\right)}\right)$$

The filter structure is very simple. The new value of output $Y_{\left(k\right)}$ depends on the last two previous values, $Y_{\left(k-2\right)}$ and $Y_{\left(k-1\right)}$, the actual input value, $U_{\left(k\right)}$, and the last old value, $U_{\left(k-1\right)}$; $K_{1}$, $K_{2}$ and $K_{3}$ are constant values that depend on refresh period and the start and stop frequency of the bandwidth, respectively. The outgoing signal from this filtering stage is provided as input using Short-Term Average/Long-Term Average (STA/LTA) method [22], suitably calibrated for the frequency bands of interest.

When an event occurs, the STA/LTA corresponding to the classification of the current event is activated. The STA/LTA algorithm detects an event when the STA/LTA ratio exceeds a fixed reference threshold. In particular, there is a threshold relating to “trigger on” and another relating to “trigger off”. When the curve is above the first threshold, it indicates the start of a significant event, while when it is below, it indicates the end of the event. “Trigger on” and “trigger off” refer to the time information about the event on the input signal. This information allows the band frequency to be determined and then sent to the server, which then processes the post-event information. Therefore, the information sent via Socket is as follows: trigger on, trigger off, Band Frequency, Station Name, PGA. Trigger on and trigger off are sent as temporal information, Band and Station Name as strings, and PGA as a floating number.

The server is connected via Socket to the ARES stations using an IP and the previously assigned port. On program start-up, the system creates as many independent processes as there are stations, starting from a configuration .csv file:

- id,ip,port,lat,lon
- STA00,147.163.105.70,3389,43.141968,13.071459
- STA01,147.163.105.76,3389,43.129766,13.066322
- STA02,147.163.105.75,3389,43.137409,13.061813

Every process has an ID that corresponds to the station’s ID, which is used to identify the station’s process (the Station Name sent by the MEMS station). When a process is killed for any reason (such as a system restart), the system recreates the process starting from the station’s assigned ID. Whenever a station triggers an alert, it sends the data to the server. The server sends an alarm if the following two conditions are satisfied:

- The number of stations triggering exceeds a threshold value (for example, 80% of stations)
- Station triggers must fall within a certain empirical time (for example, ~15 s)

**Figure 8:**
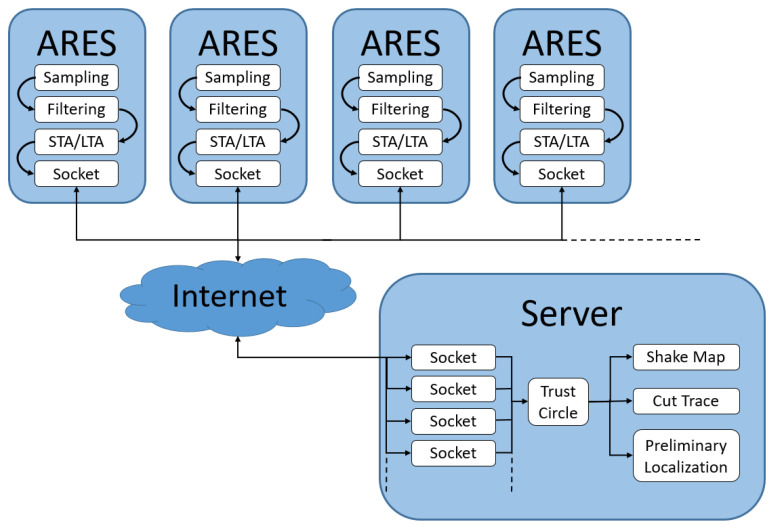
General scheme of the system.

Both the number of stations that trigger and the empirical time are configurable parameters within the server. An alarm consists of sending an email and SMS to preconfigured numbers and e-mails containing the early information about the event. The alert contains the Date, Time, Mean PGA of the Stations, and Preliminary Localization. Figure 8 shows a general scheme that summarizes the system.

## 5. The Monitoring Network

The network typology of the SHM and OE applications is a star network, in which each node is connected to a server with a point-to-point connection. This specific network was chosen because it complies with the main needs of our system, namely, flexibility, reliability, and simplicity in the addition or removal of nodes. Every node can be accessed remotely to fix possible malfunctions or update the software. The monitoring stations connect to the server via their nodes. The installation phase of stations within the edifices or locales have been accurately planned to guarantee power and connection to the nodes. SeisComP4 [23] is the dedicated software used in this application for the management of seismic data. It queries the stations and creates a daily trace file package by package. The packages are sent with a frequency of about 1 Hz. If the network fails for a few minutes, SeisComP4 contacts the station again to re-establish the connection. After the connection between server and station has been stabilized, the server requests the missing data from the station. An account of the current state of the network can be obtained via The Dude, a software program that queries different services periodically in order to monitor the status of the connectivity of nodes and stations (for details, see https://mikrotik.com/thedude, accessed on 20 April 2022). This software allows the user to access the connection statistics in order to monitor the transmission and change or make improvements to already-implemented connections, if necessary. It is possible to perform physical interventions on-site as well (for example, to change LTE antennas or move the antennas outside). The visualization of the recordings is carried out through Swarm (for more information, see https://volcanoes.usgs.gov/software/swarm/index.shtml, accessed on 20 April 2022), which is a trace viewer software for seismic data, after the data are archived on the repository by SeisComP4. The traces can be viewed in near-real time by selecting the network in the archive organization.

## 6. Camerino Network

**Figure 9:**
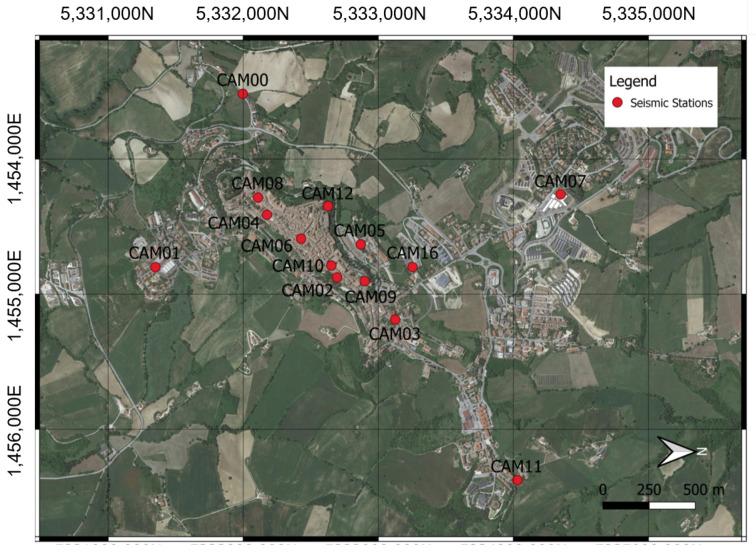
Position of the MEMS accelerometric stations deployed in the framework of the ARCH project (adapted from [3]).

**Figure 10:**
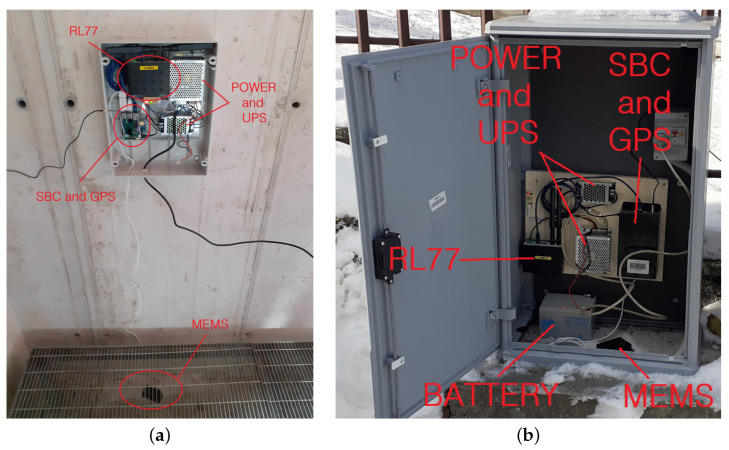
(**a**) Indoor installation with no local connection and an unstable power grid and (**b**) outdoor installation in a cabin on the road.

In Figure 9, the position of the ARCH project stations are shown on a map of the region. There are three expected installation scenarios: indoor installations with local connectivity and a permanent electrical network; indoor installations with no a local connection and an unstable power grid, as shown in Figure 10a; and outdoor installations, as shown in Figure 10b.

**Figure 11:**
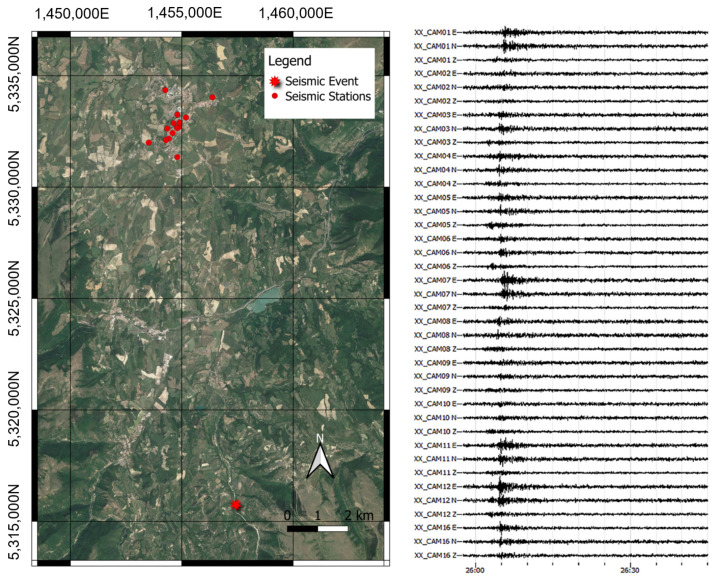
Position of the MEMS accelerometric stations with respect to the epicentre of ML3.3 earthquake, which occurred near Fiordimonte (MC) on 18 April 2021 (left panel). Accelerometric waveforms recorded by MEMS accelerometric stations during the same earthquake (right panel).

**Figure 12:**
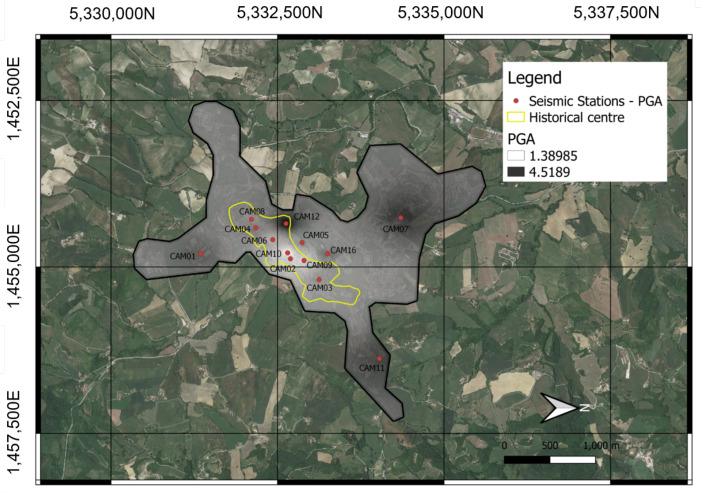
Example of shakemap obtained from the recordings carried out by the MEMS accelerometric stations during ML3.3 earthquake, which occurred near Fiordimonte (MC) on 18 April 2021 (adapted from [3]).

In all cases, the instrumentation is placed on the wall or ground using screws or dowels. Where services are always present, it is possible to simplify the system to a small box. The only drawback is that during the installation phase, it is necessary to detect the Euler angles (roll, pitch, yaw) in order to rotate the tracks using the rotation matrix. These angles must then be entered in the settings file when setting up the network and restarting the machine. On a restart, the code operations take into account the new parameters, returning the signals along the Up–Down, North–South, and East–West reference axes to the output. In the case of the example in Figure 10a, the sensor can be installed on the floor and oriented correctly with respect to the convention. The sensor is equipped with 2 m of cable in order to fix it to the ground. The SBC and the GPS are inside an IP 54 watertight box together with the power supply (UPS and Battery), including the LTE system (RL77) transmission equipment. The only limitations on the installation are the length of the power and sensor cables, which must be able to reach outside to the GPS and LTE antennas. If the GPS signal is bad, the system uses the NTP service. If the LTE signal is bad indoors, the LTE antenna must be installed outside. In the case of the example in Figure 10b, the sensor is fixed on the plinth by means of a plug. The sensitive devices have been installed and fixed on a wooden base to simplify the installation operations. The battery of the UPS system is simply placed on the plinth. Because the cabinet used here made of a plastic material, it is not necessary to bring the antennas outside. Figure 11 shows an example of the waveforms recorded by the Camerino USN following the event of 18 April 2021. As can be seen, even in an event of moderate magnitude the seismic event is clearly visible on the traces recorded by the accelerometric stations. For each of the waveforms, the peak values of the acceleration can be easily obtained, allowing the construction of urban-scale shake maps. Figure 12 is a shake map obtained from the detected event, obtained using python code; for more clarification, refer to [3,24].

## 7. Conclusions

The impact of a strong earthquake on an urban centre can be considerably reduced by an emergency management centre, which can enable timely and targeted actions in the immediate post-earthquake period. A USN consisting of a high density of stations installed in vulnerable urban centres for monitoring can provide immediate alerts and post-earthquake information summarized in maps of ground motion parameters, which can greatly improve the effectiveness of rescue operations. Centres for post-earthquake emergency management could use this information to determine their action priorities in order to minimize the loss of human life and maximize the optimal management of scarce available resources. In this work, we have presented a study of the first urban seismic network built in central Italy. Central Italy is one of the areas with the greatest seismic risk in the entire Mediterranean region, and the city of Camerino is certainly one of the municipalities with the highest seismic risk in central Italy. The high seismic risk of this municipality is linked to both its high seismic hazard and to the high vulnerability of its building heritage. Camerino was therefore an ideal historical centre for an application such as the one described in this work. The realization of the USN described in this work using low-cost materials (from a few hundred to a maximum of one thousand euros to install one station) is already an important result. While the USN at Camerino is an important technological achievement, it is a useful instrument of civil protection and research as well, representing an important tool for civil protection authorities through the provision of timely alerts that can allow for more rapid and effective mobilization of post-earthquake interventions. In addition, the implemented monitoring infrastructure can be used to test early warning algorithms for earthquakes as well as local seismic response studies. The implemented USN could be used to provide the distribution of the intensity of ground shaking due to an earthquake in a timely fashion. The resulting shaking maps, calculated in near-real time, could be used for the optimal management of priorities and the allocation of resources to achieve a significant reduction in the number of victims following an earthquake. In Figure 12, it is possible to observe how, in a very small area, the PGA (Peak Ground Acceleration) varies over a wide range of 1.9 to 4.3 cm/s^2^. This is a clear indication of side effects capable of significantly modifying the magnitude and frequency content of the earthquake. The data acquired in the future following earthquakes of different magnitudes and epicentral distance/depth will allow for full understand of the local seismic response in this territory along with any directional and non-linear effects, while the data acquired after a low-magnitude earthquake will provide important information for further seismic microzonation of the Camerino Municipality.

The limits of this application in the field of seismic monitoring are dictated by the distance of any earthquakes and their magnitude, as for events of low magnitude or high distance it is not possible to distinguish seismic signals from instrument noise. In order to solve this limitation, we intend to proceed with the development of a Digital 3D Velocimeter.

## Figures and Tables

**Table 1:** Accelerometer characteristics.

Accelerometer Characteristics	
Acceleration Measurement Max	±2 g
Acceleration Measurement Resolution	76.3 μg
Acceleration Bandwidth	497 Hz
Acceleration White Noise σ	300 μg
Acceleration Minimum Drift σ	37 μg
Acceleration Optimal Averaging Period	1037 s

## Data Availability

The data presented in this study are available on request from the corresponding author.

## Abbreviations

The following abbreviations are used in this manuscript:

USN	Urban Seismic Network
PGA	Peak Ground Acceleration
SHM	Shake Map
OE	Observatory Earthquake
STD	Standard Deviation
LTE	Long-Term Evolution
UPS	Uninterruptible Power Supply
GPS	Global Position System
PPS	Pulsations Per Second
ADC	Analog to Digital Converter
M2M	Machine to Machine
NAT	Network Address Translation
